# BPChAr—a Benzene Polycarboxylic Acid database to describe the molecular characteristics of laboratory-produced charcoal: Implications for soil science and archaeology

**DOI:** 10.1371/journal.pone.0321584

**Published:** 2025-05-14

**Authors:** Ivy Notterpek, Oliver E. Craig, Pauline Garberi, Alexandre Lucquin, Isabelle Théry-Parisot, Samuel Abiven

**Affiliations:** 1 BioArCh, Department of Archaeology, University of York, York, England; 2 Université Côte d’Azur, CNRS, CEPAM UMR, Nice, France; 3 CEREEP-Ecotron Ile De France, ENS, CNRS, PSL Université, St-Pierre-lès-Nemours, France; 4 Laboratoire de Géologie, Département de Géosciences, CNRS – Ecole normale supérieure, PSL Université, Institut Pierre Simon Laplace, Paris, France; University of Florida, UNITED STATES OF AMERICA

## Abstract

The benzene polycarboxylic acid (BPCA) method is a technique to characterise the aromaticity and aromatic condensation of pyrogenic carbon (PyC) in charred residues. As a molecular marker for polycondensed aromatic moieties, the analysis of BPCAs in archaeological contexts has great potential as a means of detecting and characterising charred residues where past fire traces are not evident. Despite the increased frequency of applications and significant developments since the method’s inception, no central database of BPCA results for modern charcoal pyrolysed under controlled laboratory conditions exists. Limited sample sizes in previous research have restricted the ability to precisely quantify the effects of combustion temperature, precursor feedstocks, pyrolysis parameters (e.g., oxygen availability), and methodological aspects (e.g., chromatography) on resultant BPCA profiles. To remedy this, we present the BPChAr database, which contains a total of 236 BPCA results on modern lab-produced charcoal. Through statistical analyses of the gathered data, we quantify the relationship between combustion temperature and resultant BPCA profiles, and construct random forest models to predict combustion temperature in unknown samples. Our findings show that additional variables hypothesised to play a role in shaping BPCA results — such as precursor feedstock type, oxygen availability during pyrolysis, and chromatographic separation method — have statistically significant implications for resultant BPCA profiles. Our analysis nuances these observations, highlighting at what charring temperatures and for what variables these concomitant parameters should be factored into the interpretation of BPCA results. Random forest models are also developed to predict precursor feedstock (hardwoods, softwoods, and grasses) in unknown samples, though further work is required to refine the accuracy of this model. The BPChAr database constitutes a fundamental tool for modern PyC research, and provides a baseline for future work aimed at employing the BPCA method in palaeoenvironmental and archaeological research.

## Introduction

Charcoal and other charred remains are routinely recovered from diverse geographic and chronological contexts. In archaeological research, charred remains in soils and sediments can be studied at a macroscopic level to reconstruct the spatial organisation of and taphonomic processes possibly affecting combustion features [1,2]. Microscopically, their taxonomic identification and analysis (anthracology) can provide crucial information regarding changing vegetal communities, anthropogenic exploitation of particular vegetal resources, and the physiological and phenological state of the wood at the time of pyrolysis (e.g., green, dried, rotten) [3–6]. Micromorphological studies can permit the observation of specific fire maintenance activities, such as trampling or sweeping [7], and help determine if a hearth was built on a prepared substrate [8]. Carbonised remains also provide a rigorous and precise means of dating archaeological assemblages through the use of radiocarbon (^14^C) dating [9–11]. Another carbon isotope, ^13^C, combined with taxonomic identification, can be analysed to refine our understandings of environmental and climatic changes signalled by taxonomic shifts in the broader charcoal assemblage [12,13].

Archaeological charcoal is thus an extremely informative media that can be used to reconstruct site chronologies, palaeoenvironmental and climatic conditions, anthropogenic fuel selection and management strategies, and possibly reveal specific pyrotechnic behaviours (e.g., the charring of wooden artefacts, see [14]). However macroscopic charcoal represents only a small fraction of the total fire-derived carbon (known as pyrogenic carbon, PyC, or black carbon, BC) generated by a combustion event, as the micro-char fraction is often quickly dispersed into the environment following a fire [15,16]. A large portion of this material has a molecular or a colloidal size [17], requiring the use of another approach to study it. However, charcoal and other charred remains are composed of complex organic compounds that pose unique analytical challenges, particularly for the application of biomolecular approaches.

As organic material such as plant biomass undergoes pyrolysis and reaches roughly 280–300 °C, the organic fraction of the fuel source is converted into volatile gases and an increasingly carbon-rich organic residue known as char. In the presence of a sufficient oxygen supply, these volatile gases and charred residues further oxidise to produce water, carbon dioxide, and the inorganic remains of the fuel source (ash). Char is therefore the result of an incomplete combustion process, and is largely composed of carbon-rich molecules arranged into aromatic rings (the most basic being benzene). When multiple aromatic rings condense, as in the pyrolysis of plant biomass, polycyclic aromatic hydrocarbons (PAHs) are formed. Under limited oxygen supply, these aromatic molecules continue to condense to form large, polycondensed aromatic structures. The term PyC is thus utilised in this work to refer to the condensed, aromatic fraction of fire-produced carbon.

**Fig 1:**
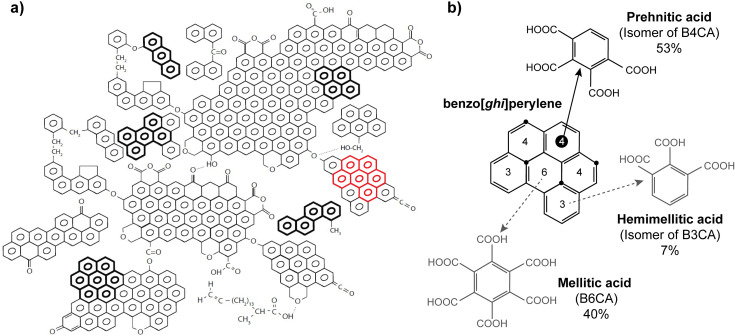
Example of BPCA production from PyC. a) Theoretical PyC molecule with highlighted PAHs to mimic edge functionalities of PyC structure, including benzo[*ghi*]perylene in red (modified from [19]); b) Example of BPCA production from benzo[*ghi*]perylene (modified from [20], with data from [21]). The numbers in the rings represent the number of carboxylic acid substitutions if that ring is oxidised to BPCA. The circled number 4 demonstrates the production of prehnitic acid, with black dots marking the positions oxidised to carboxylic acids. Only one BPCA molecule can be produced per molecule benzo[*ghi*]perylene, which is most commonly prehnitic acid (53% of total BPCA-C), followed by mellitic and hemimellitic acid (40% and 7% of total BPCA-C, respectively) (shown in grey). Total C recovery from benzo[*ghi*]perylene in the form of BPCA-C (normalised to the number of carbon atoms in each BPCA molecule) is 22.5% (± 3.9% s.d.) [20,21].

The benzene polycarboxylic acid (BPCA) method, developed in 1998 [18], employs acid oxidation to convert these polycondensed aromatic structures into aromatic monomers detectable by chromatographic techniques, and thereby serves as a molecular marker specific for polycondensed aromatic moieties (Fig 1). While a total of 12 non-nitrated BPCAs exist, BPCA analysis is concerned with those eight containing 3 or more carboxylic acid substitutions (for information on nitrated BPCAs, see [19]). These BPCAs of interest are further distinguished by their number of carboxylic acid substitutions: (i) B3CAs (trimellitic, hemimellitic, and trimesic acids), containing 3 carboxylic acid substitutions; (ii) B4CAs (pyromellitic, prehnitic, and mellophanic acids); (iii) B5CA (benzenepentacarboxylic acid); and (iv) B6CA (mellitic acid). The relative abundances of these differently carboxylated BPCAs speak to the molecular characteristics of the charred material; for instance, the heavily-substituted mellitic acid can only be extracted from the centre of aromatic clusters [19], reflecting highly aromatic and condensed PyC.

BPCA analysis provides insight into the aromaticity and aromatic condensation of PyC, including the highest temperature of treatment (HTT) at which the carbon-rich aromatic structures were formed [22–24]. Originally developed on wood char and soils [18], the BPCA method is now routinely applied to a wide range of environmental samples including charcoal, soils and sediments, water, and condensed soot particles [25,26]. Concurrent with this expansion of material types amenable to BPCA analysis was a proliferation of methodological approaches to the extraction and analysis of BPCAs [11,22,27–34], including developments spurred by concerns for non-pyrogenic OM and artificial BPCAs produced by the methodological procedure (e.g., from non-condensed materials during acid digestion) [29,35,36]. Much variability therefore exists in the literature, both methodologically and in the reporting of and interpretations drawn from BPCA results [37]. HTT remains one of the most commonly pursued variables in BPCA analyses [24], yet no consensus exists as to which BPCA relationship or ratio can best predict HTT in unknown samples. Additional parameters have been hypothesised to influence BPCA signals — such as the type of precursor biomass [25,38], oxygen availability during pyrolysis [39], and combustion duration [18] — but these variables have not been extensively quantitatively pursued in BPCA research (largely due to limited sample sizes). Lastly, while previous research has demonstrated quantitative differences between BPCA yields obtained by gas or liquid chromatographic approaches [40], such research has not been tested on larger sample sizes or expanded to the additional methodological approaches that have since emerged.

Identifying this great variability in and limitations of the available literature, we sought to create a central BPCA database of freshly charred material pyrolysed under controlled laboratory conditions in order to: (i) identify the variables that significantly influence BPCA signals; (ii) characterise the relationship of BPCA ratios and proxies with HTT; (iii) facilitate data transparency and comparability; and (iv) establish a baseline that must be considered before progressing to considerations of transformations and alterations (i.e., diagenesis) in environmental and/or ancient contexts.

The BPCA method holds great potential in archaeological research. Firstly, the PyC from which BPCAs are converted is larger, less mobile [41], highly persistent and more chemically stable [42–44] than other biomolecules frequently sought in geochemical and archaeological research (e.g., lipids, proteins, DNA). It can be applied to both macroscopic charcoal, as well as the numerous particulate charcoal fragments dispersed throughout the profile by human actions (i.e., trampling, sweeping) and/or taphonomic processes (i.e., percolating groundwater, cryoturbation). As a molecular marker for polycondensed aromatic moieties, the BPCA method may also be envisaged as a means of tracing the intensity of fire use throughout occupation phases, and of detecting combustion events where no visible charred materials remain. Lastly, the BPCA method may ultimately be employed as a proxy for the HTT reached by the charred residue. An accurate means of estimating combustion temperature has been long sought in archaeological research (e.g., [45]) to approach varied questions about fuel selection strategies and the possible function(s) of combustion features (e.g., [46,47]). Moreover, the thermal alteration experienced by the charred residue has important implications for other biomolecular and elemental methods, such as lipidomic and *δ*^13^C analyses [48–53]. To effectively employ the BPCA method in archaeological and ancient contexts, it was necessary to first create a central database of BPCA results on modern char produced under controlled laboratory conditions, and to examine the effect of and interactions between the numerous variables involved in shaping a BPCA signal.

## Materials and methods

### Data extraction and construction of the database

To construct the database, peer-reviewed publications that have applied BPCA analysis were screened for the following criteria: (i) BPCA analysis of fresh, lab-produced charcoal (i.e., not in a soil or sediment matrix, nor following degradation/incubation experiments); (ii) clear information on pyrolysis conditions and feedstock (including taxonomic nomenclature); (iii) inclusion of quality controls (i.e., internal standard recovery, details of corrections applied to raw data); (iv) data displayed or made accessible by authors involving the distribution of B3CA to B6CA. Of the approximately 50 papers screened for inclusion, a total of 14 publications acceptably met conditions for inclusion in the database, yielding a total of 236 individual entries. An additional 18 publications were identified that employ the BPCA analysis of lab-produced charcoal, but unfortunately these could not be included due to a lack of available data. One publication included in the database lacks clear taxonomic nomenclature of the tested species [39]. Full details of the publications screened for inclusion, including the reasons behind the omission of certain publications, are available in S1 Table (Sheet 2).

The variables recorded during data extraction are relayed in S1 Table (Sheet 1) and include information on the characteristics of the lab-produced charcoal, conditions of pyrolysis, organic and elemental carbon yields, methodological aspects of BPCA extraction and analysis, total and individual BPCA contents, BPCA distributions, various BPCA proxies and measurements (details in Table 1), as well as internal standard recoveries. Unfortunately gaps remain, as publications occasionally lack information such as individual BPCA yields. Consequently, BPCA isomer data could not be investigated in the present study. Wherever possible, calculations were performed with available data to maximise the number of entries for each recorded variable. Certain data points were also extracted using plot digitiser software (WebPlotDigitizer 4.7). Extracted and calculated entries are clearly indicated in the database. It is important to note that, wherever possible, individual BPCA-C contents were calculated from uncorrected total BPCA contents to account for different correction factors and ambiguity on applied corrections in the literature. In this vein, a single correction factor (2.27), as proposed by [18], was applied to obtain corrected BPCA contents. Quality control checks were performed throughout the process of data acquisition, and external validation of the extracted and calculated data was performed.

**Table 1:** BPCA ratios and proxies evaluated in this study. The variable *n* in the equation for the Average CAS refers to the number of carboxylic acid substitutions (3, 4, 5 or 6). The “expected correlations” column details the trends anticipated for the values yielded by the ratio or proxy as a function of pyrolysis temperature, with implications for aromaticity and aromatic condensation. For instance, with hotter pyrolysis temperatures (red arrow), the average CAS should increase. This is indicative of increased aromaticity (“+ arom”) and increased aromatic condensation (“+ cond”). Blue arrows indicate decreasing or cooler pyrolysis temperatures.

Ratio or proxy	Equation	Expected Correlations	Ref.
Average Number of Carboxylic Acid Substitutions (Avg. CAS)	$\Sigma (\frac{\;\%\;n-BPCA}{100}\times n)$	**↑** = ↑ (+ arom, + cond) **↓** = ↓ (‒ arom, ‒ cond)	[21]
BPCA_arom_ (‰)	$\frac{BPCA}{OC}(‰$	**↑** = ↑ (+ arom) **↓** = ↓ (‒ arom)	[23]
BPCA_cond_ (%)	$\frac{B6CA}{\Sigma \;BPCA}\;(\;\%)$	**↑** = ↑ (+ cond) **↓** = ↓ (‒ cond)	[23]
Σ(B5CA+B6CA)/ΣBPCA	$\frac{\Sigma (B5CA+B6CA)}{\Sigma \;BPCA}$	**↑** = ↑ (+ arom, + cond) **↓** = ↓ (‒ arom, ‒ cond)	[54]
Σ(B5CA+B6CA)/ Σ(B3CA+B4CA)	$\frac{\Sigma (B5CA+B6CA)}{\Sigma (B3CA+B4CA)}$	**↑** = ↑ (+ arom, + cond) **↓** = ↓ (‒ arom, ‒ cond)	[55]
B5CA/B6CA	$\frac{B5CA}{B6CA}$	**↑** = ↓ (‒ cond) **↓** = ↑ (+ cond)	[39]
B6CA/ΣB4CA	$\frac{B6CA}{\Sigma \;B4CA}$	**↑** = ↑ (+ cond) **↓** = ↓ (‒ cond)	[56]
ΣB4CA/B6CA	$\frac{\Sigma \;B4CA}{B6CA}$	**↑** = ↓ (‒ cond) **↓** = ↑ (+ cond)	[56]
ΣB4CA/B5CA	$\frac{\Sigma \;B4CA}{B5CA}$	**↑** = ↓ (‒ cond) **↓** = ↑ (+ cond)	This study
B5CA/ΣB4CA	$\frac{B5CA}{\Sigma \;B4CA}$	**↑** = ↑ (+ cond) **↓** = ↓ (‒ cond)	This study

### Statistical analysis, data sharing, and accessibility

Statistical analysis of the assembled data was performed using JMP (17.2), R and Rstudio (4.2.3). The data was first tested for normality using the Shapiro-Wilk and Anderson-Darling tests (p < 0.05), and a Spearman’s correlation test was performed. Analysis of variance (ANOVA) using the Kruskal-Wallis rank sum and post-hoc Dunn tests was utilised to investigate the statistical significance of pyrolysis temperature on quantitative BPCA outputs. Two-way ANOVA with the Benjamini-Hochberg Procedure was used to test the effect of precursor feedstock types and oxygen availability during pyrolysis on BPCA results. The effect of chromatographic separation method on BPCA results was investigated by way of Wilcoxon rank sum exact test, Wilcoxon rank sum test with continuity correction, and Welch’s two sample t-test. The exact statistical test used was determined by an R function (ANOVA, see Supplementary Information) that automatically tests the conditions for the use of each test.

Multiple methods of prediction were initially tested for the prediction of combustion temperature and precursor feedstock in unknown samples, including various regression techniques and Akaike’s Information Criterion. A random forest (RF) model utilising principal components determined via principal component analysis (PCA) was ultimately selected to reduce the dimensions considered for prediction and potential inaccuracies in future use related to different methods of reporting data (e.g., correction factors applied to individual BPCA contents). Prior to PCA analysis, redundant variables with a perfect correlation (e.g., B6CA/ΣB4CA and ΣB4CA/B6CA) were simplified such that only one variable was retained, for a total of 15 quantitative variables. Two methods of PCA analysis were tested: (i) the first, in which all entries lacking all quantitative parameters included in the model were excluded; and (ii) the second, in which missing values were estimated using an iterative PCA method (missMDA R package, see Supplementary Information). In all cases, the RF model utilises k-fold cross validation and various numbers of principal components (4–8), trees (100–1000), and mtry (i.e., the number of variables to randomly sample as candidates at each split) parameters were tested to optimise model accuracy. For qualitative estimations, classes were weighted in model training to account for differences in sample sizes.

The R code utilised for analysis, as well as codes utilised therein for specific statistical functions, are provided (S1–S4 Code). The database is freely available for downloading and consultation (10.5281/zenodo.14917459, see also S1 Table).

## Results

### Database overview: qualitative and quantitative variables

A total of 236 entries compose the database, representing a diverse assemblage of charred material for which both qualitative and quantitative variables can be explored. The qualitative variables here investigated include precursor feedstock, pyrolysis temperature (represented qualitatively for the purposes of statistical analysis), oxygen availability during pyrolysis, and chromatographic separation method (gas versus liquid chromatography).

While 14 distinct species and 14 genera are represented in the database, possible effects of precursor biomass or feedstock on BPCA signals were evaluated at the broad classificatory levels of hardwoods (*n* = 92), softwoods (*n* = 31), and grasses (*n* = 106) to increase the sample sizes available for statistical analysis. “Shells,” used to describe biochar produced from peanut shells (*Arachis hypogaea*), were excluded from analyses due to the limited sample size for this category (*n* = 7). The hardwood category is composed of two families: *Fagaceae* (e.g., *Castanea sativa*) and *Rosaceae* (e.g., *Malus*). The softwood category is composed of only one family (*Pinaceae*) and genus (*Pinus*), for which two distinct species of pine are present (*P. sylvestris* and *P. ponderosa*). The grasses category is composed of two families: *Poaceae* (e.g., *Zea mays*, *Oryza sativa*) and *Triticeae* (e.g., *Secale cereale*) (for total genera distribution by feedstock category, see S1 Fig).

Of the 236 entries here recorded, 231 were produced at temperatures ranging from 200 to 1000 °C and 5 were produced at low temperatures (60–105 °C), largely included as “drying only” controls to investigate artificial BPCA formation. To facilitate statistical analyses and examine the resolution of temperature data, four temperature categories were created: (i) “Temp 1” with 100 °C increments from 0–1000 °C; (ii) “Temp 2” with 100 °C increments from 50–850 °C with a terminal group of 850–1000 °C; (iii) “Temp 3” with 200 °C increments from 0–1000 °C; and (iv) “Temp Cat” featuring categorical grouping of low (≤ 300 °C, *n* = 68), mid (350 ≤ *x* < 700 °C, *n* = 139), and high (≥ 700 °C, *n* = 29) temperature chars. Temperature categories 1–3 are exclusive of the minimum and inclusive of the maximum value, such that the temperature grouping 200–400 °C excludes chars produced at 200 °C but includes those produced at 400 °C. Temp 3 and Temp Cat were privileged in statistical analyses to more evenly balance the number of observations across groups and to better visualize statistical tendencies.

Four possible scenarios of oxygen availability during pyrolysis were recorded in the database. Two of these are quantified — 0 (*n* = 121) and 20.5 (*n* = 86) — and describe the percent oxygen in N_2_ for samples typically produced in a pyrolysis or split tube furnace. For samples produced in a muffle or closed chamber furnace, descriptions were typically limited to “atmospheric” (*n* = 3) or atmospheric with “restricted oxygen” (*n* = 26) (i.e., using aluminium foil, ceramic crucibles, etc.). Statistical analysis of this variable thus involved treating these four scenarios as qualitative possibilities. Lastly, of the 236 entries gathered in the database, 140 were obtained by gas chromatographic methods (GC-BPCA) and the remaining 96 by liquid chromatographic methods (LC-BPCA). The detectors utilised following gas (e.g., GC-FID or GC-MS) or liquid (e.g., HPLC-C-IRMS, HPLC-DAD) chromatographic separation vary according to each study, though the majority of GC-obtained results are derived through GC-FID. The terms GC-BPCA and LC-BPCA are adopted in this work following Schneider et al. [40] to statistically evaluate the impact of the chromatographic separation method on BPCA results, and group the various detection methods employed in each study. For the number of individuals associated with each category described above, see S2 Table (Sheet 1).

The quantitative variables here evaluated largely concern BPCA outputs, ratios, and proxies as presented in Table 1. The number of entries and summary statistics for all quantitative variables here studies are relayed in Table 2.

**Table 2:** Summary statistics for all quantitative variables recorded in the database.

Variable	*n*	Min	Max	Mean	SD
Pyrolysis temperature (°C)	236	60.00	1000.00	453.06	187.89
Corrected BPCA (g BPCA-C/kg OC)	235	1.02	793.59	321.02	163.85
Uncorrected BPCA (g BPCA-C/kg OC)	235	0.45	349.60	141.42	72.18
B3CA-C (g/kg OC)	169	0.00	59.81	10.44	8.25
B4CA-C (g/kg OC)	169	0.00	108.30	38.03	20.01
B5CA-C (g/kg OC)	169	0.00	108.76	48.10	24.51
B6CA-C (g/kg OC)	208	0.00	243.11	56.96	45.31
B3CA (%)	170	0.00	100.00	8.22	9.15
B4CA (%)	170	0.00	57.60	26.60	10.29
B5CA (%)	170	0.00	80.07	32.14	10.20
B6CA (%)	209	0.00	97.70	34.09	19.96
Average CAS	170	3.00	5.98	4.90	0.40
BPCA_arom_	235	0.05	34.96	14.14	7.22
BPCA_cond_	209	0.00	97.70	34.09	19.96
Σ(B5CA+B6CA)/ΣBPCA	169	0.00	1.28	0.66	0.16
Σ(B5CA+B6CA)/Σ(B3CA+B4CA)	168	0.00	187.68	4.28	15.77
B5CA/B6CA	187	0.02	8.60	1.30	0.83
B6CA/ΣB4CA	167	0.00	174.00	3.41	14.65
ΣB4CA/B6CA	167	0.00	10.18	1.22	1.30
ΣB4CA/B5CA	169	0.00	5.46	0.89	0.55
B5CA/ΣB4CA	167	0.18	13.68	1.52	1.48

BPCA ratio values are determined using individual BPCA-C contents.

### How does pyrolysis temperature affect BPCA profiles?

**Fig 2:**
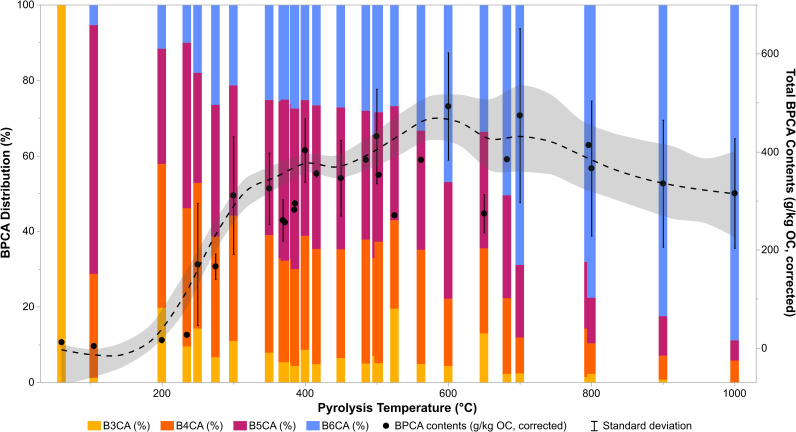
Total BPCA contents and relative distributions as a function of pyrolysis temperature. BPCA distribution (%, left) and total corrected BPCA contents (g BPCA-C/kg OC, right) as a function of pyrolysis temperature (°C) for all values with all measurements (*n* = 170). The black points represent the mean of total BPCA contents (corrected), fit with a smoothing spline (lambda: 0.05), and each error bar is constructed using one standard deviation from the mean.

Fig 2 shows that as charring or pyrolysis temperature increases, total BPCA yields (g/kg OC) gradually increase from an average of 6.1 ± 3.6 at temperatures below 200 °C (*n* = 5) to reach a maximum of approximately 470.4 ± 131.6 at 700 °C (*n* = 10). Average total BPCA contents, which are reflective of the aromaticity of the charred material, do not further increase and tend to taper off at higher temperatures (acknowledging the large standard deviations observed for high temperature chars due to comparatively limited sample sizes). Concurrently, the proportion of mellitic acid to all other BPCAs (described by the BPCA_cond_ index) steadily increases with increasing pyrolysis temperature. These values increase from 7.7 ± 6.6 in chars produced below 200 °C (*n* = 5) to approximately 25.9 ± 5.8 in chars produced between 300 and 500 °C (inclusive, *n* = 119). BPCA_cond_ values then leap to 47.2 ± 7.8 at 600 °C (*n* = 24), and reach a maximum of 86.9 ± 7.2 at 1000 °C (*n* = 6). Fig 2 also demonstrates that the relative proportion of B3CA is relatively inconsistent from 100 to 800 °C, suggesting that B3CA yields may not be very reliable for the interpretation of BPCA results. However, together, total BPCA contents and the relative proportions of individual BPCAs appear to have a clear and diagnostic relationship to HTT.

**Fig 3:**
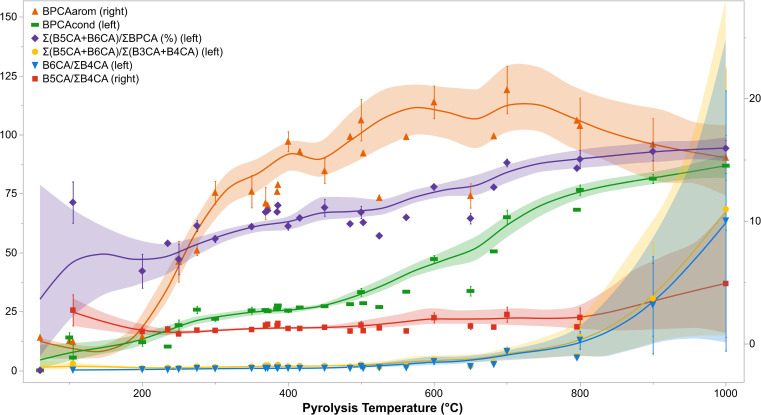
BPCA ratios and proxies with a generally positive correlation with increasing pyrolysis temperature. Note the axes associated with each variable (left, right) as listed in the legend. Σ(B5CA+B6CA)/ΣBPCA was converted to a percent for sake of representation. Each point represents the population mean and each error bar represents one standard error from the mean. The splines present have an associated lambda value of 0.05.

**Fig 4:**
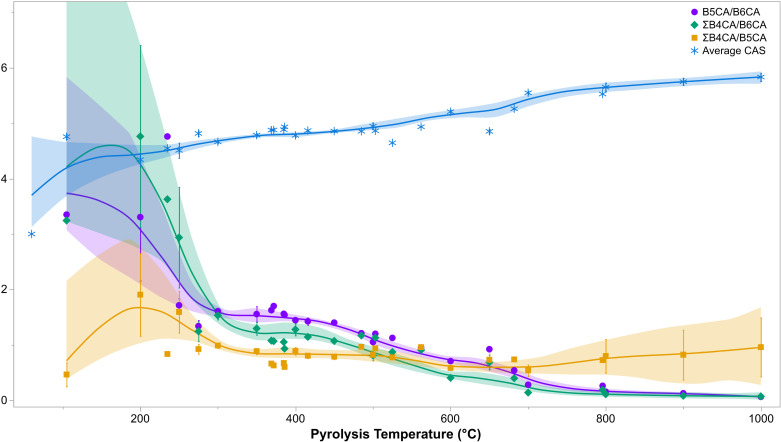
BPCA ratios and proxies with a generally negative correlation with increasing pyrolysis temperature (with the exception of average CAS). Each point represents the population mean and each error bar represents one standard error from the mean. The splines present have an associated lambda value of 0.05.

Each ratio and proxy listed in Table 1 can be evaluated in relation to pyrolysis temperature. Fig 3 presents the distribution of ratios and proxies with a generally positive correlation to increasing pyrolysis temperature (with the exception of the Average CAS, shown in Fig 4 for sake of scale). As the BPCA_arom_ index is directly proportional to total BPCA contents, the distribution of this variable is identical to that of uncorrected BPCA contents shown in Fig 2. The BPCA_cond_ and relative proportion (%) of B5CA and B6CA to total BPCA contents follow a similar slope, and converge for high temperature chars. The proportions of B6CA/ΣB4CA and of heavily substituted [Σ(B5CA+B6CA)] to lightly substituted [Σ(B3CA+B4CA)] BPCAs increase exponentially at high charring temperatures, from values of approximately 1 at 500 °C to 66 at 1000 °C. A similar pattern is demonstrated by the proportion of B5CA to B4CA, though the increase with high pyrolysis temperatures is less marked and fluctuations are observed for low temperature chars.

The Average CAS has a positive trend with increasing pyrolysis temperature (Fig 4), with values increasing from approximately 4.3 ± 0.3 in chars produced at 200 °C (*n* = 6) to 4.8 ± 0.2 at 400 °C (*n* = 26), 5.2 ± 0.1 at 600 °C (*n* = 20), and 5.8 ± 0.1 at 1000 °C (*n* = 4). The remaining BPCA ratios in Fig 4 have a negative trend with increasing pyrolysis temperature. The values of ΣB4CA/B6CA are highest for low temperature chars, and converge with those of B5CA/B6CA at 300 °C. B5CA/B6CA values are slightly higher than those of ΣB4CA/B6CA after 300 °C until approximately 800 °C, after which both ratios yield values of approximately 0.1 to 0.2 (Fig 4), reflecting the heavily condensed nature of chars produced at these temperatures. The ratio of ΣB4CA/B5CA follows a more irregular distribution, with a peak of approximately 1.9 at 200 °C that descends to approximately 0.8 from 350–550 °C and rises slightly for high temperature chars (0.96 at 1000 °C) (Fig 4).

**Fig 5:**
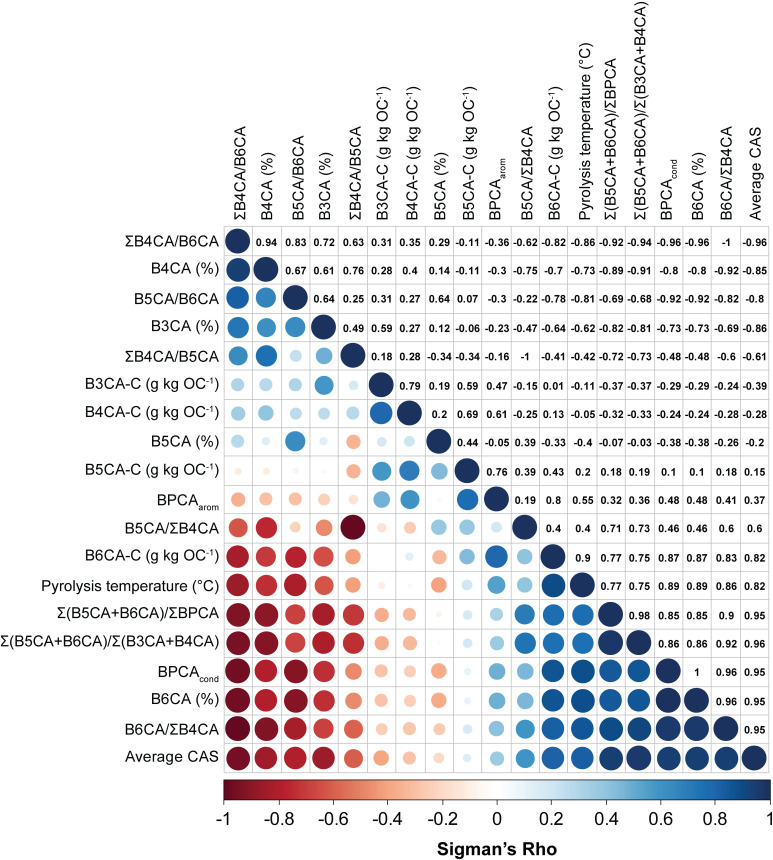
Spearman rank correlation plot and associated correlation coefficients of quantitative BPCA outputs. Lower left: shading and circle size correspond to the importance of the correlation. Upper right: correlation coefficients yielded by the analysis.

All of the quantitative variables recorded in the database fail the Shapiro-Wilk and Anderson-Darling tests for normality (p < 0.05). As such, a Spearman’s correlation test was performed to evaluate the statistical significance of any monotonic relationships between them (Fig 5). Pyrolysis temperature has the strongest correlation with BPCA_cond_ and B6CA % (0.89), followed by B6CA/ΣB4CA (0.86) and its inverse ΣB4CA/B6CA (-0.86), the Average CAS (0.82), and lastly B5CA/B6CA (-0.81). Total BPCA contents (not shown in Fig 5 but represented by BPCA_arom_) and heating duration (not shown) did not demonstrate a strong (> 0.5 or < -0.5) correlation with any of the BPCA ratios or proxies. Similarly, BPCA contents of benzene tri- to penta-carboxylic acids did not yield a strong correlation with any BPCA ratios or proxies, with the exception of the BPCA_arom_ index for B4CA-C (0.61) and B5CA-C (0.76) (Fig 5). B6CA-C contents are, in contrast, strongly correlated with the vast majority of quantitative variables. Interestingly, B5CA (%) yielded the weakest correlation coefficients of all BPCAs described by their relative abundance (Fig 5). Together, this correlation matrix suggests that BPCA outputs recorded as ratios or relative abundances rather than absolute quantities are better suited for the interpretation of BPCA results, and furthermore, that ratios or proxies reflective of aromatic condensation rather than aromaticity are more salient for the estimation of combustion temperature.

To determine at what temperatures statistically significant differences in quantitative BPCA outputs exist, numerous statistical tests (e.g., ANOVA, Kruskal-Wallis rank sum and post-hoc Dunn tests) were performed wherein quantitative variable outputs were evaluated as a function of pyrolysis temperature utilising the four temperature groupings described previously. While statistically significant differences were observed in all cases, the ability to distinguish different temperature groups was weakest with 100 °C increments (Temp 1 and 2). The results of these tests on 200 °C increments (Temp 3) are shown in Table 3, and those for low, mid, and high temperature chars (Temp Cat) in S2 Table (Sheet 2).

**Table 3:** Results of Kruskal-Wallis rank sum test and Dunn’s post-hoc test for all quantitative BPCA outputs as a function of pyrolysis temperature, categorised in 200 °C increments. The degrees of freedom for each Dunn’s test is 4. Temperature ranges with the same letter are not statistically distinct (p < 0.05). The p-values in the final column test the null hypothesis that no differences among the temperature categories exist.

Variable	Temperature Range (°C)					Significance
	0–200	200–400	400–600	600–800	800–1000	
Corrected BPCA (g BPCA-C/kg OC)	a	b	c	c	bc	χ^2^ = 63.655 p = 4.94e-13
Uncorrected BPCA (g BPCA-C/kg OC)	a	b	c	c	bc	χ^2^ = 63.655 p = 4.94e-14
B3CA-C (g/kg OC)	a	b	bc	ac	a	χ^2^ = 50.415 p = 2.957e-10
B4CA-C (g/kg OC)	a	b	b	a	a	χ^2^ = 58.147 p = 7.106e-12
B5CA-C (g/kg OC)	a	b	c	a	a	χ^2^ = 65.492 p = 2.027e-13
B6CA-C (g/kg OC)	a	b	c	d	d	χ^2^ = 137.03 p < 2.2e-16
B3CA (%)	a	a	b	bc	c	χ^2^ = 62.987 p = 6.829e-13
B4CA (%)	a	a	b	c	c	χ^2^ = 79.689 p < 2.2e-16
B5CA (%)	a	a	a	b	b	χ^2^ = 52.106 p = 1.311e-10
B6CA (%)	a	b	c	d	d	χ^2^ = 151.08 p < 2.2e-16
Average CAS	a	a	b	bc	c	χ^2^ = 98.712 p < 2.2e-16
BPCA_arom_	a	b	c	c	bc	χ^2^ = 63.655 p = 4.94e-13
BPCA_cond_	a	b	c	d	d	χ^2^ = 151.03 p < 2.2e-16
Σ(B5CA+B6CA)/ ΣBPCA	a	a	b	bc	c	χ^2^ = 85.154 p < 2.2e-16
Σ(B5CA+B6CA)/ Σ(B3CA+B4CA)	a	a	b	c	c	χ^2^ = 78.387 p = 3.825e-16
B5CA/B6CA	a	a	b	c	c	χ^2^ = 109.13 p < 2.2e-16
B6CA/ΣB4CA	a	b	c	d	d	χ^2^ = 104.33 p < 2.2e-16
ΣB4CA/B6CA	a	b	c	d	d	χ^2^ = 105.6 p < 2.2e-16
ΣB4CA/B5CA	ab	a	b	b	ab	χ^2^ = 22.866 p = 0.0001347
B5CA/ΣB4CA	ab	a	b	b	ab	^χ^2^^ = 24.725 p = 5.713e-05

Of the 20 tested variables, 5 (B6CA-C, B6CA %, BPCA_cond_, B6CA/ΣB4CA, and ΣB4CA/B6CA) were able to distinguish chars produced in the 0–200, 200–400, 400–600, and 600–800 °C temperature ranges. An additional 4 [B5CA-C, B4CA %, Σ(B5CA+B6CA)/Σ(B3CA+B4CA), and B5CA/B6CA] were able to differentiate chars produced at 200–400, 400–600, and 600–800 °C. Lastly, B5CA-C and the 3 variables related to total BPCA contents (BPCA_arom_, corrected and uncorrected values) were able to distinguish chars produced at 0–200, 200–400, 400–600 °C. While no variable was able to statistically distinguish between chars produced at 600–800 °C and those produced at 800–1000 °C, 11 of 20 variables were able to distinguish low temperature chars produced at 0–200 °C and 200–400 °C. S2 Fig illustrates the examples of the BPCA_arom_ and BPCA_cond_ indices. At the larger categorical grouping low, mid, and high temperature chars, 12 of 20 variables were able to statistically identify each temperature grouping. Of the 8 remaining variables, 5 were able to distinguish low from mid-high temperature chars (BPCA corrected and uncorrected, BPCA_arom_, ΣB4CA/B5CA, B5CA/ΣB4CA), two were able to distinguish high from low-mid temperature chars (B3CA-C and B5CA %), and B5CA-C was able to distinguish mid from low and high temperature chars.

### Do feedstock and/or pyrolysis conditions affect BPCA profiles?

A goal of this research was to explore the potential for feedstock and/or pyrolysis conditions to affect BPCA profiles, and three variables were statistically analysed to this end: (i) precursor feedstock category (hardwoods, softwoods, grasses); (ii) oxygen availability during pyrolysis (% O in N_2_); and (iii) combustion duration (minutes at HTT).

#### Possible feedstock effects.

The possible effect of precursor biomass type on BPCA signals was explored by means of two-way ANOVA with the Benjamini-Hochberg Procedure on all quantitative variables for low, mid, and high temperature chars among the precursor feedstock categories of hardwoods, softwoods, and grasses [see S2 Table (Sheet 3)]. Of the 16 statistically significant outcomes (p < 0.05), 7 were observed within low temperature chars, 2 within mid-temperature chars, and 6 in high temperature chars.

In low temperature chars, a total of 4 variables demonstrated statistically significant outcomes in respect to precursor feedstock, including the relative proportion (%) of B3CA and B5CA, ΣB4CA/B6CA, and ΣB4CA/B5CA. Significant differences (p < 0.01) were observed for B3CA (%) between grasses and hardwoods, with hardwoods yielding higher relative B3CA amounts than grasses. No significant differences were observed in B3CA (%) amounts for softwoods. Conversely, for B5CA (%) amounts, both grasses and softwoods yielded higher relative B5CA amounts than hardwoods (p < 0.001 and 0.05, respectively). For ΣB4CA/B6CA, hardwoods yielded statistically significant higher values than both grasses and softwoods (p < 0.05 and 0.01, respectively). Lastly, for ΣB4CA/B5CA, hardwoods yielded statistically significant higher values than both grasses and softwoods (p < 0.0001, p < 0.01 respectively).

The only statistically significant results observed for mid-temperature chars concern B5CA-C contents, where grasses were distinct from (p < 0.05) and yielded lower B5CA-C contents than both hardwoods and softwoods. In high temperature chars, statistically significant differences were exclusively related to variables concerning aromatic condensation, specifically: Σ(B5CA+B6CA)/Σ(B3CA+B4CA), B6CA/ΣB4CA, and B6CA (%, also reflected by BPCA_cond_). It is worth noting however that only 1 softwood entry exists for high temperature chars, thereby limiting the full extent of statistical comparisons possible for this category. For both Σ(B5CA+B6CA)/Σ(B3CA+B4CA) and B6CA/ΣB4CA, these differences were notable (p < 0.0001) between grasses and hardwoods, with grasses yielding higher values than hardwoods. For B6CA (%), no statistically significant difference was found between grasses and hardwoods, but both were statistically distinct from (p < 0.001) and yielded higher values than softwoods (acknowledging the fact that this category holds only 1 entry).

#### Oxygen availability during pyrolysis.

To investigate the possible effect of oxygen availability during pyrolysis on BPCA results, two-way ANOVA with the Benjamini-Hochberg Procedure was conducted on all quantitative variables for low, mid, and high temperature chars among the air composition categories “0,” “20.5,” “atmospheric,” and “atmospheric (restricted oxygen)” per the data gathered in the database [see S2 Table (Sheet 4)]. While standard atmospheric conditions are relatively consistent with 20.5% oxygen, this distinction was maintained for the sake of precision and clarity. Of the 41 statistically significant outcomes (p < 0.05), 32 were observed within low temperature chars, 7 within mid-temperature chars, and 2 in high temperature chars.

**Fig 6:**
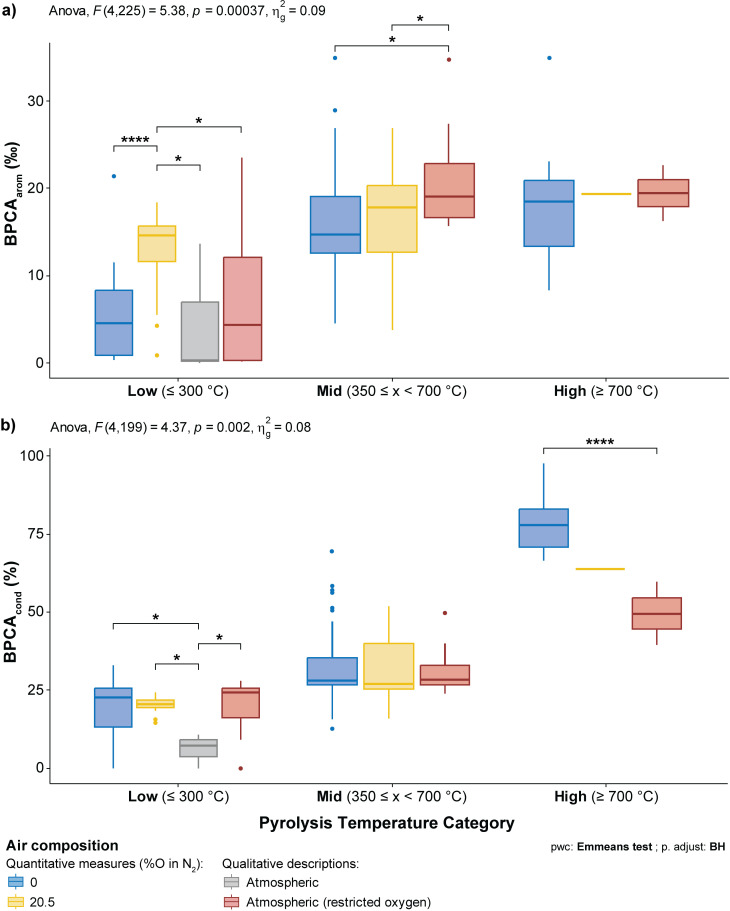
Two-way ANOVA results of a) BPCAarom (‰) and b) BPCAcond (%) for the analysis of oxygen availability during pyrolysis in low, mid, and high temperature chars. Associated sample sizes for BPCA_arom_ were as follows: low (*n* = 68), mid (*n* = 138), and high (*n* = 29). Associated sample sizes for BPCA_cond_ were as follows: low (*n* = 64), mid (*n* = 116), and high (*n* = 29). Asterisks denote statistically significant differences with the following associated p-values: **** < 0.0001, *** < 0.001, ** < 0.01, * < 0.05.

Quantitative variables with statistically significant differences according to air composition in low temperature chars include: individual B3CA, B4CA, and B5CA contents; B5CA (%) and B6CA (%); the BPCA_arom_ and BPCA_cond_ indices (Fig 6); corrected and uncorrected BPCA contents; and lastly ΣB4CA/B6CA. Statistically significant differences were observed between all possible combinations of air composition, with the majority between 20.5 and atmospheric, followed by 0 and 20.5. The most statistically significant differences (p < 0.0001) were all observed in measures of individual BPCA contents (B3CA-C, B4CA-C, B5CA-C, total BPCA contents, and BPCA_arom_) between the 0 and 20.5 categories of air composition, with oxygenated chars yielding higher values than those pyrolysed only in N_2_.

In mid-temperature chars, a total of four variables demonstrated statistically significant differences according to air composition including B5CA and total BPCA contents (corrected, uncorrected, BPCA_arom_). For B5CA-C, these differences were observed between the 0 and 20.5 categories of air composition (p < 0.0001), with oxygenated chars again yielding higher values than those produced under an inert atmosphere. For total BPCA contents (corrected, uncorrected, BPCA_arom_), significant differences (p < 0.05) were observed between both 0 and 20.5 with atmospheric (restricted oxygen), with the latter yielding higher BPCA contents (Fig 6).

In high temperature chars, only relative amounts of B6CA (%, BPCA_cond_) had statistically significant differences (p < 0.0001) according to air composition. This difference was observed between the 0 and atmospheric (restricted oxygen) categories, with chars produced under an inert atmosphere yielding greater relative amounts of B6CA than those produced under atmospheric (restricted oxygen) conditions (Fig 6). Examples of this analysis are shown in Fig 6 for the BPCA_arom_ and BPCA_cond_ indices, and full results for all variables are available in S2 Table (Sheet 4).

#### Heating duration.

Heating duration was recorded as the minutes maintained during pyrolysis at HTT, and 12 outcomes were obtained: 15 (*n* = 1), 30 (*n* = 4), 60 (*n* = 21), 120 (*n* = 13), 180 (*n* = 68), 240 (*n* = 14), 300 (*n* = 94), 600 (*n* = 1), 720 (*n* = 3), 1440 (*n* = 1), “cooled after reaching set temperature” (treated as 0, *n* = 14), and “n/a” (flash pyrolysis, *n* = 2). To explore this variable, heating duration was examined within two categories of charcoal — those produced at 300 °C and 400 °C — as these categories had the largest sample sizes with variation in heating duration parameters. In charcoal produced at 300 °C, individual BPCA yields slightly increased with charring time while the relative proportion of BPCAs (%) remained relatively stable, with the exception of a slight decline in B5CA (%) amounts. In 400 °C charcoal, individual BPCA amounts remained relatively stable with a slight decline in B5CA-C contents with increased charring time. The relative amounts of BPCAs also varied, with B4CA (%) amounts slightly increasing and B5CA (%) amounts slightly decreasing with increased charring time. Further research is needed to clarify any possible effect of heating duration on resultant BPCA profiles.

### Does chromatographic separation method affect BPCA profiles?

To evaluate the effect of chromatographic separation method on quantitative BPCA outputs, numerous statistical tests were performed on all entries (*n* = 236), as well as on low (*n* = 68), mid (*n* = 139), and high (*n* = 29) temperature chars [see S2 Table (Sheet 5)]. For all entries irrespective of pyrolysis temperature, statistically significant (p < 0.05) differences as determined by Wilcoxon rank sum test with continuity correlation between results obtained by gas (GC-BPCA) or liquid (LC-BPCA) chromatography were observed for 5 of the 20 tested variables: B3CA-C, B5CA-C, B3CA %, ΣB4CA/B5CA, and B5CA/ΣB4CA. Two variables, B6CA-C and B4CA (%), yielded statistically significant differences between GC- or LC-BPCA within each category of low, mid-, and high temperature chars but did not yield significant results when regarding the dataset as a whole.

For low temperature chars, a total of 9 variables demonstrated statistically significant differences between GC- and LC-BPCA as determined by Wilcoxon rank sum test with continuity correlation. These include all variables measuring total or individual BPCA contents, B3CA %, B4CA %, and the BPCA_arom_ index. GC-BPCA results yielded higher total BPCA contents, higher relative B3CA contents (%), and lower relative B4CA contents (%) than LC-BPCA in these low temperature chars (S3 Fig).

Seven variables demonstrated statistically significant differences between GC- and LC-BPCA in mid-temperature chars, as determined by Wilcoxon rank sum (exact and with continuity correction) and Welch’s two-sample t-test, including: B3CA-C (and B3CA %), B5CA-C, B6CA-C, B4CA %, ΣB4CA/B5CA, and B5CA/ΣB4CA. For individual BPCA contents and the relative proportion of B3CA (%), GC-BPCA results yielded higher results than LC-BPCA; conversely, B4CA (%) values were higher in mid-temperature chars with LC-BPCA than GC-BPCA (S3 Fig). Accordingly, ΣB4CA/B5CA values were higher in LC-obtained results.

For high temperature chars, statistically significant differences were observed in 10 of 20 variables, including B4CA-C, B6CA-C, and B4CA (%). Here, statistical significance was observed in ratios and proxies not previously significant, including the Average CAS, Σ(B5CA+B6CA)/ΣBPCA, Σ(B5CA+B6CA)/Σ(B3CA+B4CA), B6CA/ΣB4CA, and ΣB4CA/B6CA. While total BPCA contents were not statistically significant between the two chromatographic separation methods (p = 0.07), LC-obtained results do appear generally greater and this lack of statistical significance may be due to a high outlier for GC-BPCA (S3 Fig). B4CA-C, B4CA (%), and B6CA-C results were greater in LC-BPCA than GC-BPCA. Proxies reflective of aromatic condensation such as the Average CAS, Σ(B5CA+B6CA)/ΣBPCA, Σ(B5CA+B6CA)/Σ(B3CA+B4CA), and B6CA/ΣB4CA were higher in GC-obtained results than LC-obtained results (S3 Fig).

### Random forest predictive models: combustion temperature and precursor feedstock

#### Estimating combustion temperature in unknown samples.

**Fig 7:**
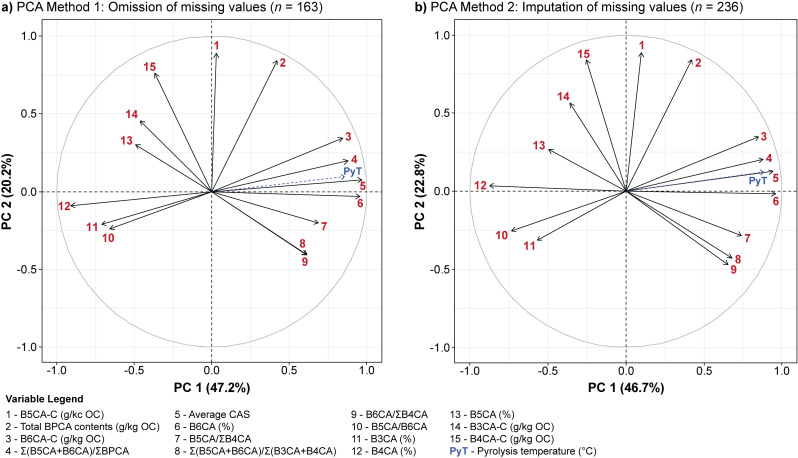
Principal component analysis of the BPChAr dataset for random forest prediction. Loading plot of principal component analysis of 15 quantitative variables (removing redundant variables with a correlation of 1.00) for a) all entries with all data (*n* = 163), and b) all entries with missing values calculated via iterative principal component analysis (*n* = 236). Pyrolysis temperature is projected onto the loading plots as a supplementary variable.

A major goal of this work was to assess the potential to predict the maximum temperature obtained during pyrolysis of an unknown charcoal sample. In the first PCA method (see “Statistical analysis, data sharing, and accessibility”) using all entries with all data (*n* = 163), the first two principal components explain 67.4% of variance in the data (PC 1: 47.2%, PC 2: 20.2%) while PC 3 and 4 explain 12 and 10.3% of variance in the data, respectively (Fig 7). These first four principal components all have associated Eigenvalues greater than 1. The second PCA method utilising iterative PCA to estimate missing values (*n* = 236) yielded a similar loading plot to the first PCA, with the first two principal components accounting for 69.5% of variance in the data (PC 1: 46.7%, PC 2: 22.8%) followed by PC 3 (11%) and PC 4 (8.8%) (Fig 7). As in the first model, the first four principal components have Eigenvalues greater than 1.

Utilising these two PCA models, RF algorithms were created and run to predict combustion temperature in unknown samples. In the first set of RF models, combustion temperature was estimated quantitatively with confidence intervals providing a minimum and maximum HTT range at 95% and 68% confidence. In the second set of RF models, combustion temperature was estimated qualitatively using categories of 200 °C increments, which were weighted in model training to account for differences in sample sizes. A statistical summary of these results is available in S2 Table (Sheet 6).

In both manners of estimating combustion temperature, the RF model trained on data wherein missing values were omitted (PCA 1) was slightly more accurate than the RF model utilising imputed values (PCA 2). For the quantitative estimation of combustion temperature (confidence level = 0.95), the lowest error rates for PCA 1 were obtained using 6 principal components with a mtry value of 6 (RMSE: 58.83 ± 25.21, R^2^: 0.90 ± 0.09, MAE: 45.94 ± 19.08), compared to 7 principal components with a mtry value of 7 for PCA 2 (RMSE: 60.14 ± 26.90, R^2^: 0.89 ± 0.15, MAE: 46.63 ± 20.08). For the qualitative estimation of combustion temperature, PCA 1 yielded an accuracy rate of 81.07 ± 17.83% (error: 0.19, kappa: 0.70 ± 0.27) utilising 7 principal components with a mtry value of 1, and PCA 2 yielded an accuracy rate of 79.74 ± 14.64% (error: 0.20, kappa: 0.66 ± 0.25) utilising 8 principal components and a mtry value of 3. While the best model parameters will differ each time the RF is run due to the random nature in which training groups are selected, the qualitative predictions provided by these models permit the estimation of combustion temperature in unknown samples with an accuracy rate of approximately 80%.

#### Reconstructing precursor feedstock in unknown samples.

A final set of RF models was constructed to predict precursor feedback at the level of hardwoods, softwoods, and grasses in unknown modern charcoal samples. As shell entries were excluded from consideration, the principal component analysis utilised to develop the model was slightly different from that used in the estimation of combustion temperature. Two PCA methods were tested, as before, to compare model accuracy when missing values are omitted (*n* = 157) or imputed (*n* = 229). Various numbers of principal components, trees, and mtry parameters were tested to optimise model accuracy, and the precursor feedstock categories were weighted to account for differences in sample sizes in the training dataset.

Accuracy levels for the prediction of precursor feedstock were weaker than that of temperature, around 60%. In this instance, the second PCA approach utilising imputed values yielded higher accuracy rates and better prediction performance (accuracy: 59.02 ± 22.66%, error: 0.41, kappa: 0.30 ± 0.38) than that in which missing values were omitted (accuracy: 57.86 ± 24.16%, error: 0.42, kappa: 0.26 ± 0.43). Additional tests were performed to evaluate if accuracy levels for the prediction of precursor feedstock could be improved when pyrolysis temperature was taken into account, yet these results were inconclusive and further research is needed to develop the possible prediction of precursor feedstock in unknown samples. The results of all RF models can be found in S2 Table (Sheet 6).

## Discussion

### Does the analysis of the BPChAr database support previous findings?

#### Combustion temperature and the estimation of HTT in unknown samples.

The strong correlation of BPCA profiles with HTT has been noted since 2010 [22], in which the same feedstock (*Castanea sativa* wood) was pyrolysed at different temperatures from 200 to 1000 °C. It was found that total BPCA-C (g/kg OC) contents gradually increased from 200 °C to peak at about 700 °C, followed by a slight decline at high temperatures [22]. The BPCA aromaticity index (BPCA_arom_) mirrors this trend [23]. Concurrently, the proportion of mellitic acid to all other BPCAs (BPCA_cond_) was found to steadily increase with increasing pyrolysis temperature [22,23]. This study supports these observations, with a peak in BPCA_arom_ and total BPCA contents at 700 °C followed by a plateau or slight decline in values at temperatures above 700 °C. The BPCA_cond_ index was found to increase consistently with increasing pyrolysis temperature to reach a maximum at 1000 °C.

This plateau in BPCA contents at high temperatures may be due to one or both of the following scenarios: (i) at high charring temperatures, the number of aromatic rings in PyC reaches a maximum (such that aromaticity stabilises) while aromatic rings continue to condense (such that aromatic condensation continues to increase); and (ii) the BPCA digestion protocol is unable to fully digest the highly aromatic, condensed structures found in high temperature chars. As a slight decline in aromaticity has been observed at very high temperatures [22], methodological shortcomings likely factor into this observation. However, it is also possible that at high charring temperatures, PyC reaches a point wherein aromaticity is saturated but the aromatic structures can continue to condense. Future research evaluating the potential to optimise the digestion protocol for high temperature chars could aid in the resolution of this question.

Despite the strong correlation of BPCA contents and distributions with pyrolysis temperature, previous research has not yet established an empirical relationship to estimate HTT in samples with an unknown combustion temperature. A single ratio (B5CA/B6CA) has been utilised to reconstruct past fire traces, as the study of Wolf et al. [39] identified three dominant fire regimes and their associated average combustion temperatures via a literature review: grass and forest ground fires (285 ± 143 °C), shrubland fires (503 ± 211 °C) and domestic fires (797 ± 165 °C). The study [39] found that natural charcoal originating from forest ground fires yielded B5CA/B6CA values of 1.3 to 1.9, from grass fires of 0.8 to 1.4, and from domestic fires of < 0.8. Charcoal originating from shrubland fires could not be distinguished, likely due in part to the overlap between the temperature ranges associated with this fire regime and the others identified. B5CA/B6CA values have thus been mobilised to infer past fire regimes in several studies [e.g., 57–61], using these values as references, though interpretations are generally restricted to tracing fire intensity through time.

The analysis of the data here gathered supports the strong correlation of B5CA/B6CA values with pyrolysis temperature; however, B5CA/B6CA had the fifth most significant Spearman correlation coefficient with pyrolysis temperature following the BPCA_cond_ index, B6CA/ΣB4CA, ΣB4CA/B6CA, and the Average CAS (Fig 5). The range of B5CA/B6CA values encountered in the database is also significant (~8.58), largely due to a single high outlier. When this outlier is removed, all B5CA/B6CA values reported for natural and domestic fires [39] are observed (0.02–4.74). The ratio of B5CA/B6CA may therefore not be the most accurate means of reconstructing combustion temperature or past fire regimes in unknown samples, and additional ratios may be worthy of future exploration.

Our analysis of all published data and of the constructed random forest models has demonstrated that the accurate prediction of HTT is improved by utilising not only multiple quantitative BPCA outputs, but also multivariate components as determined by PCA. We acknowledge, however, that the random forest models developed in this work for the prediction of HTT should undergo validation in future research through double-blind tests on experimental samples with known pyrolysis temperatures. Relating combustion temperatures to past fire regimes requires additional interpretive leaps, which are complicated by the large overlap in temperature ranges documented in natural fires as well as the innumerable variables which impact a combustion event (e.g., precursor feedstock, atmospheric conditions, oxygen availability, combustion duration). Accurate HTT estimation thus requires consideration of these concomitant variables in addition to the full suite of BPCA ratios and proxies. In this vein, this work has sought to establish what effect, if any, precursor feedstock category, oxygen availability, and combustion duration play in the formation of BPCA profiles.

#### Influence of precursor feedstock.

Previous work has hypothesised a possible difference in BPCA results according to precursor biomass, as PAH formation varies according to the relative abundance of lignin and holocellulose (cellulose and hemicellulose) in precursor feedstocks and appears positively correlated with lignin abundance [62–67]. Softwood contains a higher proportion of lignin to holocellulose compared to hardwoods or grasses [27,68]. Lignin is also more thermally stable than cellulose or hemicellulose, degrading over a larger range and at higher temperatures [69–71]. If precursor feedstock types were to impact resultant BPCA yields, it is likely that these differences would only be observable at lower pyrolysis temperatures where lignin and holocellulose components are not fully degraded and still influence the chemical structures of the aromatic compounds produced by pyrolysis. However, previous BPCA research has been unable to distinguish precursor biomass types within charcoal samples. The identification of PyC derived from charcoal versus condensates has been performed using the ratio of B5CA/B6CA, but PAH analysis was needed to distinguish feedstock ‘types’ and could only do so at the order of hardwoods, softwoods, and grasses [25].

Our research has shown that statistically significant differences in BPCA results according to precursor feedstock categories (hardwoods, softwoods, and grasses) exist among low, mid, and high temperature chars for a total of 9 variables (S2 Table, Sheet 3). The highest number of statistically significant variables were observed among low temperature chars, supporting previous hypotheses that any precursor feedstock effects are likely most salient at low pyrolysis temperatures due to the presence of plant biopolymers (e.g., lignin, holocellulose). In low temperature chars, it appears that hardwoods demonstrate lower amounts of aromatic condensation (reflected, for instance, in ΣB4CA/B6CA) than both softwoods and grasses. In mid-temperature chars, statistically significant differences were only observed for B5CA-C amounts (S2 Table, Sheet 3), wherein grasses yielded lower amounts than both hardwoods and softwoods. In high temperature chars, the majority of statistically significant variables were related to aromatic condensation (S2 Table, Sheet 3). Many of these were cases in which both grasses and hardwoods yielded higher measures of aromatic condensation than softwoods; however, given that only 1 softwood sample was available for high temperature chars, further research is needed to confirm these observations. Between grasses and hardwoods, it appears that grasses demonstrate a higher level of aromatic condensation, as reflected by Σ(B5CA+B6CA)/Σ(B3CA+B4CA) and B6CA/ΣB4CA amounts.

We have also shown that the prediction of precursor feedstock at the level of hardwoods, softwoods, and grasses using a RF model is possible. Accuracy rates are not satisfactory, however, which may be due to concomitant variables and the significant influence of pyrolysis temperature on the constitution of BPCA profiles. Furthermore, the grasses and hardwood categories were composed of only two families and the softwood category of one. This highlights a greater limitation of the literature, in which only a handful of taxa (e.g., *Castanea sativa*, *Oryza sativa*) are well-represented. We thus encourage the expansion of BPCA research to include a greater diversity of taxa, routine measurements of lignin and holocellulose abundances, and to test additional low charring temperatures to further clarify the possible effects of lignin and holocellulose composition on BPCA profiles.

#### Oxygen availability during pyrolysis.

PAH formation is greatest in oxygen-limited conditions [63,72–74] as an ample oxygen supply increases the efficiency of combustion, leading to complete rather than incomplete combustion. Accordingly, charred material from oxygen-starved combustion events may yield a greater amount and more heavily substituted BPCAs than that from well-oxygenated combustion events. Previous research has highlighted this potential effect, as the B5CA/B6CA values obtained by Schneider et al. [22] from charcoal produced under an inert atmosphere were lower (c. 0.3) than those obtained by Wolf et al. [39] from charcoal produced in an oxygenated atmosphere. As BPCA-C contents are normalised to the TOC content of the sample, well-oxygenated conditions provoking a more complete combustion will also lower TOC yields and have an impact on resultant BPCA values.

Our work has shown that statistically significant differences in obtained BPCA results according to atmospheric conditions during pyrolysis are greatest for low temperature chars, and that oxygenated conditions at low charring temperatures actually resulted in higher individual and total BPCA contents than oxygen-starved combustion conditions (Fig 6). This observation held true for B5CA-C contents in mid-temperature chars, with oxygenated conditions yielding higher values than chars produced in an inert atmosphere (S2 Table, Sheet 4). Differences for total BPCA contents were less notable, however, as only atmospheric (restricted oxygen) conditions yielded higher values than both the 0 and 20.5 air composition categories (which were themselves statistically indistinguishable). No statistically significant differences in the relative distribution of BPCAs were observed for mid-temperature chars, but in low temperature chars, atmospheric pyrolysis conditions were statistically distinct from all other atmospheric conditions for B5CA (%) and B6CA (%) amounts (Fig 6). In high temperature chars, significant differences in B6CA (%) amounts were observed, with atmospheric (restricted oxygen) conditions yielding lower BPCA_cond_ amounts than chars produced under an inert atmosphere (Fig 6). No statistically significant differences in B5CA/B6CA values were observed in any temperature category (S2 Table, Sheet 4), challenging the significance of previous observations [22,39].

This study thus confirms that oxygen availability during pyrolysis has an effect on resultant BPCA profiles. It is interesting to note that oxygenated conditions yielded higher total BPCA contents (reflective of aromaticity) at low charring temperatures, and lower B6CA (%) contents (reflective of aromatic condensation) at high charring temperatures. Understanding which variables are impacted by atmospheric composition during pyrolysis, and at what charring temperatures these differences emerge, constitutes a significant advancement for future research.

#### Heating duration.

The work of Glaser et al. [18] on pine wood charred at 300 °C for varying amounts of time found that the yield of individual BPCAs increased with increasing charring time; however, the relative distribution of BPCAs was not significantly different (with the exception of 15-minute charring time). This pattern was mostly upheld in the BPChAr database for chars produced at 300 °C, but was not upheld for chars produced at 400 °C. However, entries with combustion durations above 300 minutes were limited and further research is needed to expand these sample sizes for accurate statistical comparison. These preliminary results indicate that the possible effect of heating duration on BPCA results may depend not only on pyrolysis temperature, but also on precursor feedstock, oxygen availability, and other concurrent pyrolysis parameters. Further research is ultimately needed to clarify if and how combustion duration impacts PyC aromaticity and aromatic condensation as reflected in BPCA results.

#### Chromatographic separation method.

Previous research [40] has observed greater analytic variability (std. dev. from the mean) and systematically lower BPCA yields for GC-BPCA measurements compared to LC-BPCA for chars produced in the intermediate temperature range of 275–700 °C, with LC-obtained measurements yielding an average of 1.5 ± 0.3 times higher total BPCA contents for charcoal produced in this range. In this database, LC-obtained total BPCA contents are on average 1.06 times higher than those obtained by GC-BPCA for charcoal produced between 275 and 700 °C, and 1.07 times higher using the mid-temperature char category here employed (350 ≤ *x* < 700 °C, *n* = 139), thereby lowering the 1.5 correction factor proposed by [40]. We have additionally observed that while GC-BPCA yields are higher than LC-BPCA for low temperature chars, LC-BPCA has higher yields than GC-BPCA for high temperature chars – further complicating the use of a single correction factor. No statistically significant differences between GC- and LC-BPCA were observed for the proportion of B5CA or B6CA (BPCA_cond_), though significant differences were observed for the proportion of B3CA (low and mid-temperature chars) and B4CA (mid and high temperature chars). This is contrary to the work of Schneider et al. [40], in which no qualitative biases were observed for BPCA distributions obtained by the two methods, and highlights an important caveat that must be considered in future work.

The statistical analysis of GC- and LC-obtained results for all ratios and proxies appear to suggest that GC-BPCA overestimates B5CA and mellitic acid contributions to high temperature chars compared to LC-BPCA. More likely, however, is the possibility that the relatively small amounts of B3CAs and B4CAs in high temperature chars are lost in the more intensive sample preparation required for GC-BPCA. As no studies have detailed systematic losses of particular BPCAs due to methodological or other parameters (e.g., sample HTT), further experimental research is necessary to investigate this hypothesis and, if supported, identify procedural points that may account for these losses (e.g., evaporation during oxidation). It is also possible that the derivatization of heavily carboxylated BPCAs (particularly mellitic acid) is incomplete in high temperature chars, resulting in lower B6CA recovery and skewing resultant relative contributions. Chromatographic separation methods, including their implications for PyC extraction and sample preparation, are therefore a fundamental factor to consider when comparing BPCA results obtained by liquid and gas chromatographic approaches. Additional methodological parameters (e.g., digestion time, conventional versus microwave-assisted digestion [34,36]) should be considered in future studies.

### Extension to archaeological materials and matrices

This study was motivated by the intent to apply BPCA analysis to archaeological charcoal and charred material in soils and sediments. With a better understanding of BPCA signals and the variables that influence them on modern charcoal, we have characterised the baseline to which archaeological and ancient material can be compared. To do so, several important factors must be taken into consideration. Firstly, naturally-pyrolysed materials will have increased heterogeneity in their aromatic structures compared to material charred under controlled pyrolysis conditions. These heterogeneous aromatic molecules are then subjected to diverse post-depositional and taphonomic processes, which can alter their aromatic composition and resultant BPCA profile. The pyrogenic origins of BPCAs must also be demonstrated, alongside considerations of BPCA production from petrogenic sources and non-pyrogenic organic matter. The extension of BPCA analysis, and particularly the RF models here developed, to archaeological materials and matrices requires careful consideration of these issues.

Despite the considerable persistence of PyC in environmental matrices over millennia [e.g., 75,76], research has demonstrated that particulate PyC in soils and sediments can leach to release dissolved PyC that is then transported to aquatic environments [77,78]. The work of [79,80] found that PyC losses to leaching were generally greater in surface soils than in subsurface soils. The solubility of char also increases with ageing, with the work of Abiven et al. [17] finding that aged char leachates contained 40–55 times more condensed structures than that of fresh char. Once solubilised, dissolved PyC is more susceptible to photodegradation, with the most condensed aromatic components and B6CA being particularly affected [81–83]. Dissolved PyC is also susceptible to biodegradation, for instance by bacteria [84] and remineralisation [85]. As these processes are likely interrelated, careful considerations are due for the analysis of dissolved PyC in aquatic environments (see [37]).

The particulate PyC that remains in soils and sediments is affected by these diagenetic pathways, particularly those of movement and biodegradation. The particle size, porosity, and density of PyC affect its potential fragmentation and movement within the soil/sediment profile [86] through processes such as cryoturbation [41] or during rainfall events [15]. In addition to environmental factors [87], the degree of movement is affected by precursor feedstock [15,88], pyrolysis temperature [79,89], and characteristics of the soil/sediment (e.g., aggregate stability) [15,16,90,91] — all of which merit further investigation. For instance, high temperature chars are typically more porous and have higher specific surface areas than low temperature chars [86], which has implications for the fragility of the charcoal to physical fragmentation, its adsorptive properties, and susceptibility to remineralisation, microbial attack, and oxidation [92–96]. Research has demonstrated that oxidised PyC is more mobile and susceptible to leaching than fresh PyC [16,17]. However, the oxidation of PyC through ageing (increasing soil residence time) increases its surface reactivity and interactions with the soil mineral phase [16]. These organo-mineral reactions ultimately assist the long-term retention of PyC in soils [97,98], rendering soil the largest global PyC pool [99].

Particulate PyC in soils and sediments can also be degraded by bacteria, non-lignolytic fungi, and microbial enzymes. At a molecular level, the less aromatic and more weakly condensed components of PyC are likely more bioavailable and conducive to microbial breakdown than heavily condensed aromatic clusters, and this microbial attack may in turn render them more easily solubilized [17,100]. This degradation pathway would lead to the selective enrichment of more heavily condensed aromatic clusters, yielding a greater proportion of heavily carboxylated BPCAs (B5CA and B6CA) relative to those produced by the initial, non-degraded PyC input. However, less condensed aromatic clusters with more functional side chains may be more amenable to interactions with the soil mineral phase that increase their stability and retention than heavily condensed aromatic clusters with less functional side chains [101]. The microbial biodegradation of PyC is further mitigated by soil aggregate stability, as stable soil aggregates that form around or bind to chars will limit their mobility [15], reduce their surface area, and protect them from microbial attack. Research has also shown that the microbial mineralisation of PyC is quite slow [44], with high temperature charcoal more resistant to mineralisation processes [80,102,103].

The degradation of PyC in environmental matrices is thus conditioned by a host of factors. In the immediate depositional environment, these include characteristics such as soil depth, particle size, aggregate stability, mineral composition, pH, and the abundance and diversity of SOM and soil microbial communities. At a slightly larger scale, these include environmental factors such as mean annual rainfall amounts and the presence of wet-dry or freeze-thaw cycles. Much research has been dedicated to better understand PyC cycling in open systems such as forests [43,104,105], permafrost-affected forest and tundra soils [41,76,106], and grasslands [107]. PyC cycling has also been studied as a result of anthropogenic practices, such as slash-and-burn techniques [108, see also 109] and rice straw burning in rice paddy management [56]. It is important to note that the mobility of and diagenetic processes affecting PyC may be influenced by some of the characteristics here examined, namely pyrolysis temperature (HTT) and precursor feedstock type.

Non-pyrogenic sources of BPCAs should also be carefully considered in the extension of BPCA analysis to unknown archaeological materials and matrices. These non-pyrogenic sources include petrogenic (petroleum) sources, and non-pyrogenic organic matter. BPCAs have been identified in petrogenic sources including lignite, crude oil, and bituminous coal [110–113]. While petrogenic interference cannot be identified by BPCA analysis alone [112], it can be identified through complementary analyses including PAH [114] and compound specific isotopic analysis [111, see also 115]. The consideration of petrogenic carbon input is particularly important in contexts likely to contain petrogenic source material, such as urban soils [114] and aquatic sediments [116]. Extensive research has been conducted on the potential of non-pyrogenic organic matter with cyclic carbon forms (e.g., chlorophyll derivatives) or non-condensed aromatic structures (e.g., lignin) to produce artificial BPCAs through the methodological process. This method-induced BPCA formation can occur through acid catalysis, as was hypothesised for HCl pretreatment [29], and/or through the oxidation of these cyclic/non-condensed aromatic structures during nitric acid digestion.

Numerous materials with PyC-like structures have been investigated for their potential to produce BPCAs, including: polyphenol polymerisation products, Maillard reaction productions, and microbial biomass [18]; *Aspergillus niger* fungal C, *Penicillium citrinum*, melanoidin C, and microbial biomass [29]; chlorophyllin and β-carotene [35]; 4,9-dihydroxyperylene-3,10-quinone (DHPQ) derivatives [117]; PyC-free vegetal biomass [31,34–36,100]; and lastly humic substances and kerogen [31,100,117,118]. Method-induced BPCA formation has been disproven for some of these materials (e.g., *Penicillium citrinum*), acknowledged for others (e.g., *Aspergillus niger*, DHPQ derivatives), and remains contested for humic substances (for a review of the results of these studies, see [37]). The formation of artificial BPCAs from uncharred plant biomass, likely originating from lignin, has been rendered absent or negligible through the limitation of sample OC contents — though a precise “threshold,” after which these contributions become problematic, remains unclear [35,36,100]. Lastly, recent research has demonstrated that naturally oxidised biomass can also produce non-pyrogenic aromatic structures capable of producing BPCAs [119]. It is therefore paramount to assess and confidently demonstrate the pyrogenic origins of PyC in the studied materials, which has been raised by several studies [118–120].

These many factors must be considered for the interpretation of BPCA yields from archaeological charcoal and sediments. We posit that the BPCA yields obtained for archaeological charcoal are indicative of the HTT experienced by the charred material; this should not be confounded with other measures of heat such as radiative heat output. When applied to soils and sediments, the measured BPCA profile likely reflects the average HTT to which the micro-charcoal and particulate PyC found therein were exposed. However, the random forest models developed in this work cannot be directly transposed to archaeological charcoal and sediments. Determining how degradative pathways may be affecting BPCA results in archaeological and ancient contexts is critical to the interpretation of these results. For instance, the preferential degradation of less condensed aromatic clusters through microbial reworking or leaching could lead to an overestimation of B5CA and B5CA in the resultant BPCA profile, resulting in a higher HTT prediction than that actually experienced by the charred material. Conversely, future research may find that interactions with the mineral phase of soils and sediments, which are more likely for (less condensed) aromatic clusters featuring side chains, preferentially preserve these fractions of PyC and thus selectively degrade more heavily condensed aromatic clusters — thereby lowering HTT predictions.

The PyC content of the sample can also significantly impact BPCA results, with higher PyC levels limiting the effects of method-induced BPCA formation from non-pyrogenic organic matter. In charcoal material and PyC-dense soils such as terra preta [108,121], any error from non-pyrogenic BPCA production is proportionally insignificant given the predominance of PyC. As PyC levels decrease, the potential interference of non-pyrogenic organic matter and method-induced BPCA formation increases, such that a soil with low PyC contents can have significant non-pyrogenic interference. For instance, while SOM may generally contain 10‒30% aromatic carbon [122], PyC contributions to SOM on the order of 4% have been documented in fire-protected environments [107,122,123] and as low as 2% in organic-rich deposits such as varved lake sediments [124]. In these latter cases, the risk of misinterpretation through method-induced BPCA formation from non-pyrogenic organic matter sources is high.

In light of these methodological concerns, the origins of PyC, PyC contents, and taphonomic processes affecting PyC preservation in the depositional environment must be well characterised for the accurate interpretation of BPCA results. The application of complementary methods such as elemental and isotopic analysis [e.g., 111], PAH analysis [e.g., 114], and solid state ^13^C Nuclear Magnetic Resonance [e.g., 125] can be utilised to identify and characterise some of these interfering, non-pyrogenic materials (specifically petrogenic BC sources). However, future research is ultimately needed to better understand non-pyrogenic interference (including method-induced BPCA formation) and PyC degradation processes in different depositional environments.

As our understanding of PyC cycling, preservation, and resultant BPCA profiles continue to evolve, it was critical to establish a baseline of BPCA profiles for modern charcoal produced under controlled laboratory conditions and to characterise the variables that most influence these profiles. This baseline serves as a necessary reference to which archaeological and ancient environmental samples can be compared and interpreted in light of the geochemistry of the depositional environment and further PyC diagenesis studies. We encourage the expansion of this database to include BPCA results obtained in laboratory and open-air diagenesis/mobility studies [15,16,44,79,100,126], natural samples following wildfires [24,105], as well as archaeological and palaeoenvironmental contexts [56,57,60,61,75,124,127,128]. The incorporation of this data is crucial to rigorously investigate the impact of environmental aging on resultant BPCA profiles, and to better understand the mechanisms for PyC persistence over geological timescales.

## Conclusions and future perspectives

This work constitutes the first central database of BPCA results obtained for modern charcoal produced in-laboratory under controlled pyrolysis conditions. Our analysis of the gathered data corroborates the significant effect of HTT on BPCA signals. The strong correlation of BPCA profiles with HTT has here permitted the construction of random forest models utilising principal component analysis to predict HTT (both quantitatively and in 200 °C increments) in unknown char samples. We have demonstrated that statistically significant differences in BPCA results according to precursor feedstock (hardwoods, softwoods, grasses), atmospheric conditions during pyrolysis (e.g., oxygenated or inert atmosphere), and chromatographic separation method do exist — detailing between what categories, variables, and charring temperatures these differences emerge. Oxygen availability during pyrolysis and chromatographic separation method had a particularly notable impact on resultant BPCA profiles, while that of precursor feedstock type was comparatively weaker. Nevertheless, we were able to construct random forest models to predict precursor feedstock in unknown samples and accuracy levels can be improved through the expansion of reference materials. Further research is also needed to expand sample sizes for the investigation of combustion duration on BPCA results. Parsing out which concomitant variable may be influencing resultant BPCA profiles either in a single study or in a comparison of studies thereby requires careful consideration of the charred materials at hand, the conditions of their production, and means of their detection and quantification.

As a molecular marker for condensed aromatic moieties, the BPCA method holds great promise as a means of tracing diachronic fire histories (whether natural or anthropogenic) and of detecting past combustion events where macroscopic charcoal fragments have not been preserved. Going forward, we advocate for the reporting of BPCA-C results normalised to dry sample weights as well as OC contents, with clear details of any corrections applied during quantification to aid data transparency and comparability. In this vein, we encourage the reporting of individual BPCA isomer yields, as there are likely isomer-specific behaviours and trends that have yet to be addressed by the research community. Precise details of the characteristics of the sample and of the pyrolysis conditions employed will also further our ability to investigate additional variables possibly affecting BPCA profiles. Additionally, actualistic combustion experiments and incubation studies (with the reporting of the initial BPCA yields) will assist applications to unknown samples from ancient paleoenvironmental and archaeological contexts. We further advocate for the expansion of BPCA research to better reflect the diversity of charred plants, organic materials (e.g., foodcrusts), and combustion conditions encountered in the environmental and archaeological record. Ultimately, while this database constitutes a first step towards the accurate and effective application of BPCA analysis to archaeological and ancient contexts, it is a significant advancement necessary to synthesise current understandings and facilitate data transparency and accessibility in future research.

## Supporting information

S1 Fig Frequency of each genera by feedstock category (hardwood, softwoods, grasses, shell) and scientific family. Labels denote the number of entries for that genus.
(TIF)

S2 Fig Results of Kruskal-Wallis rank sum test and Dunn’s post-hoc test for a) BPCA_arom_ and b) BPCA_cond_ as a function of pyrolysis temperature, categorised in 200 °C increments. The degrees of freedom for each Dunn’s test is 4. Temperature ranges with the same letter are not statistically distinct (p < 0.05).
(TIF)

S3 Fig Comparison of chromatographic separation method. Distribution of GC-obtained (blue) and LC-obtained (red) BPCA results for: a) BPCA_arom_; b) Σ(B5CA+B6CA)/ΣBPCA; c) B3CA-C (g/kg OC); d) B3CA (%); e) B4CA-C (g/kg OC); f) B4CA (%); g) B5CA-C (g/kg OC); h) B5CA (%); i) B6CA-C (g/kg OC); and j) B6CA (%). Results are separated by pyrolysis temperature category for low (≤ 300 °C), mid (350 ≤ x < 700 °C), and high (≥ 700 °C) temperature chars. All conclusions regarding the statistical significance of differences between the two chromatographic separation methods are relayed in S2 Table (Sheet 5).
(TIF)

S1 Table BPChAr database. **Sheet 1: Useful information.** Table of Contents and useful information. **Sheet 2: Bibliography.** Bibliography of publications screened and selected for inclusion in the database. **Sheet 3: BPChAr database. Sheet 4: Treated data.** Pre-treated data for seamless application of the R code found in the Supporting Information.
(XLSX)

S2 Table Additional information and results of statistical tests. **Sheet 1: Qualitative variables.** Number of entries according to the qualitative variables here studied. **Sheet 2: Pyrolysis temperature.** Kruskal-Wallis rank sum test and Dunn’s post-hoc test results for all quantitative BPCA outputs as a function of pyrolysis temperature, categorised by low (≤ 300 °C, *n* = 68), mid (350 ≤ x < 700 °C, *n* = 139), and high (≥ 700 °C, *n* = 29) temperature chars. The degrees of freedom for each Dunn’s test is 2. Temperature categories with the same letter are not statistically distinct (p < 0.05). **Sheet 3: Precursor feedstock.** Results of two-way ANOVA with the Benjamini-Hochberg Procedure on all quantitative variables for low, mid, and high temperature chars among the precursor feedstock categories of hardwoods, softwoods, and grasses. **Sheet 4: Oxygen availability during pyrolysis.** Results of two-way ANOVA with the Benjamini-Hochberg Procedure on all quantitative variables for low, mid, and high temperature chars among the air composition categories “0,” “20.5,” “atmospheric,” and “atmospheric (restricted oxygen).” **Sheet 5: Chromatographic separation method.** Summary of statistical tests to investigate the effect of chromatographic separation method (GC- or LC-BPCA) for all, low, mid, and high temperature chars. The statistical test used was automatically determined in each case by the R code according to the sample size and results of the variable for the Shapiro-Wilk and Bartlett tests of normality. Statistically significant p-values are indicated in bold. **Sheet 6: Random Forest results.** Summary statistics for random forest predictive models testing various numbers of principal components, trees, and mtry parameters with PCA method 1 (omission) and 2 (imputation) for the treatment of missing values. In each case, the model with highest accuracy is indicated in bold.
(XLSX)

S1 Code R code utilised for the analysis of the BPChAr database. All functions and statistical tests presented in this work can be reproduced utilising this code and the data in S1 Table (Sheet 4). Supplementary codes for proper functioning are provided in S2–S4 Code.
(R)

S2 Code ANOVA 2. Integrated code for ANOVA and related tests for non-normally distributed data.
(R)

S3 Code Correlations. Integrated code for correlation functions (e.g., Spearman correlation).
(R)

S4 Code Fonction predictions. Integrated code necessary for the random forest models.
(R)
