# Supplementary figures and images for: BPChAr—a Benzene Polycarboxylic Acid database to describe the molecular characteristics of laboratory-produced charcoal: Implications for soil science and archaeology
Source: PLoS One. 2025 May 14;20(5):e0321584. doi: 10.1371/journal.pone.0321584 (PMC12077725; doi:10.1371/journal.pone.0321584)

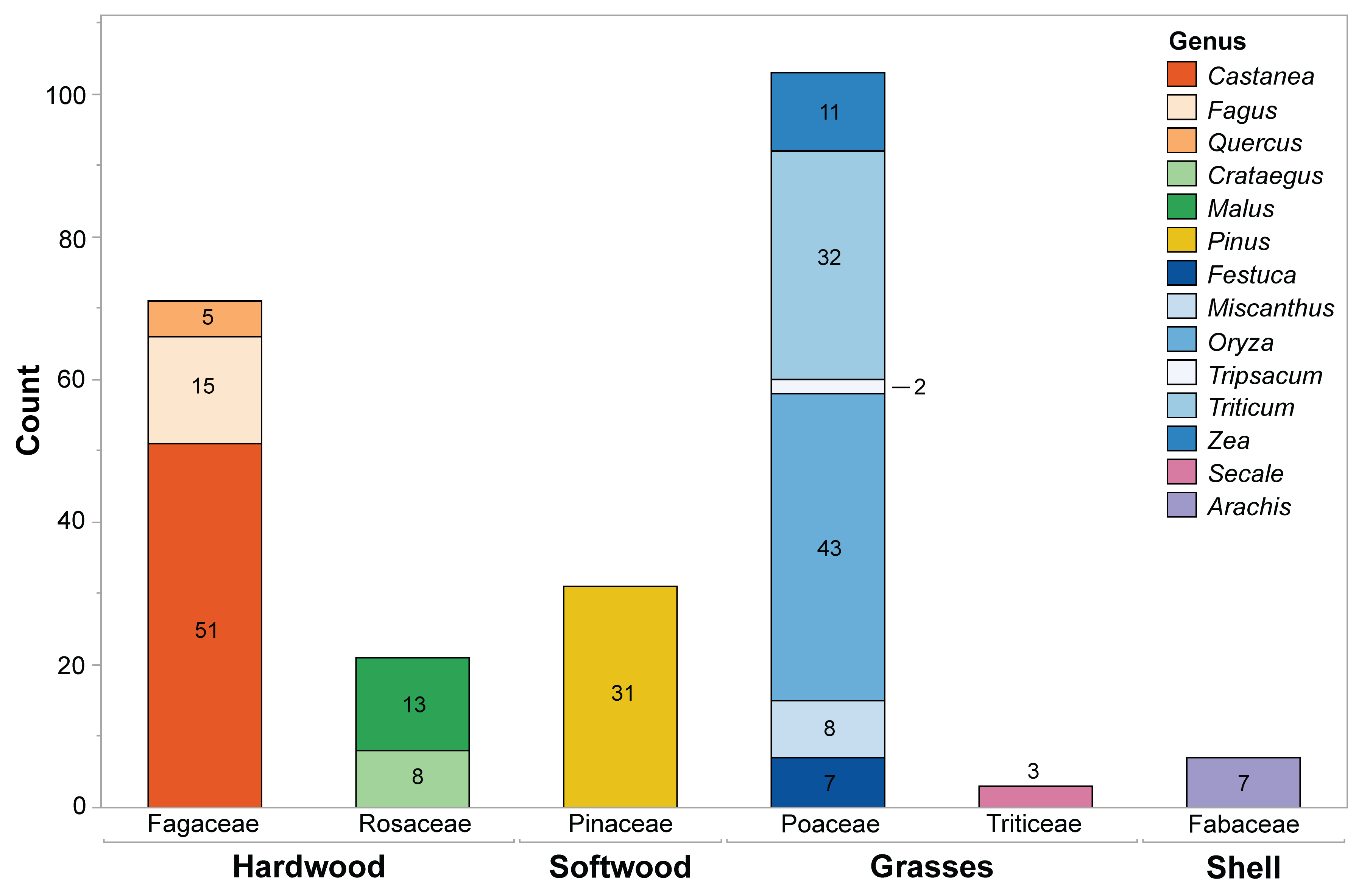

Supplement: S1 Fig — Labels denote the number of entries for that genus. (TIF) [file pone.0321584.s001.tif]

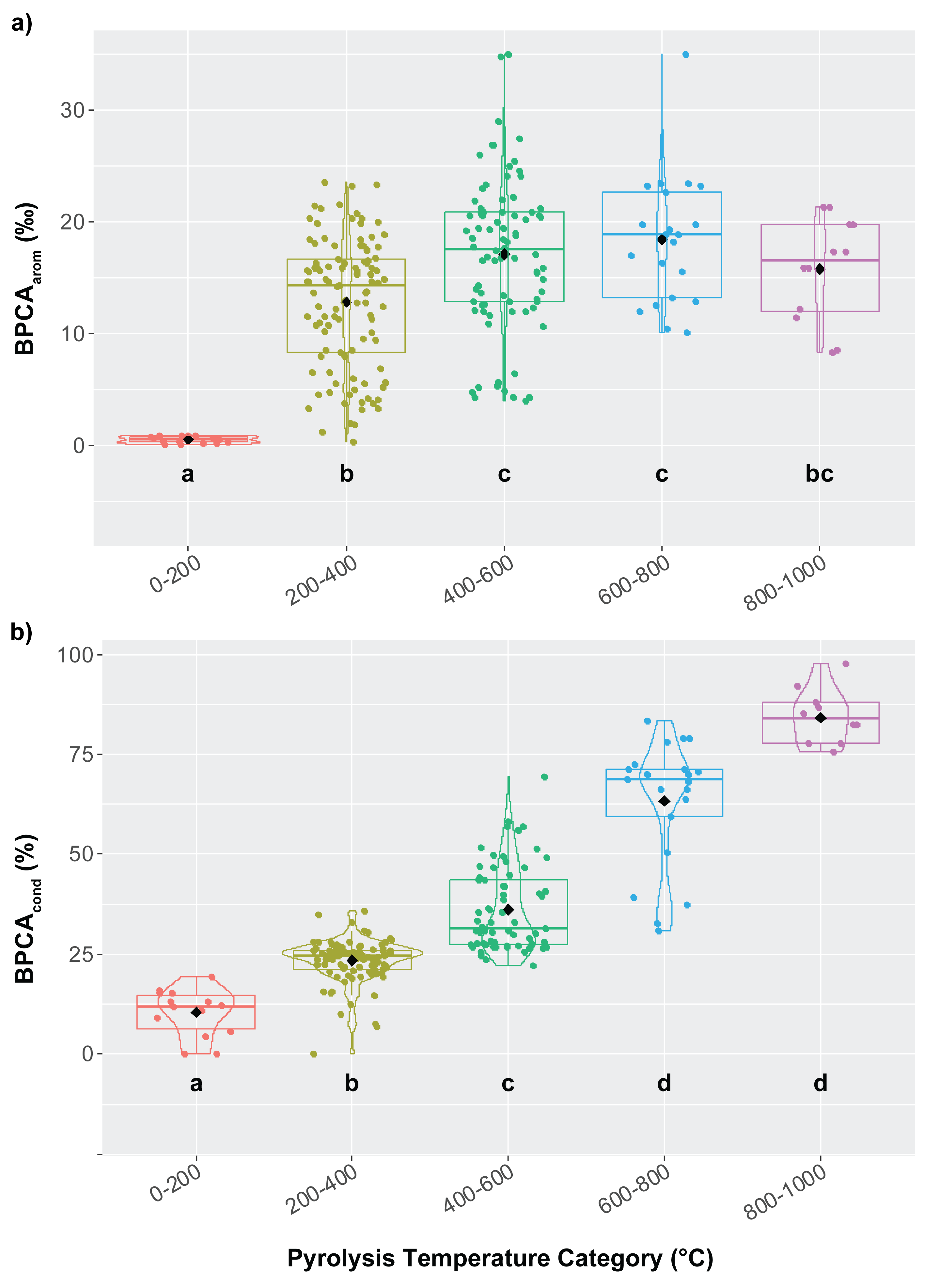

Supplement: S2 Fig — The degrees of freedom for each Dunn’s test is 4. Temperature ranges with the same letter are not statistically distinct (p < 0.05). (TIF) [file pone.0321584.s002.tif]

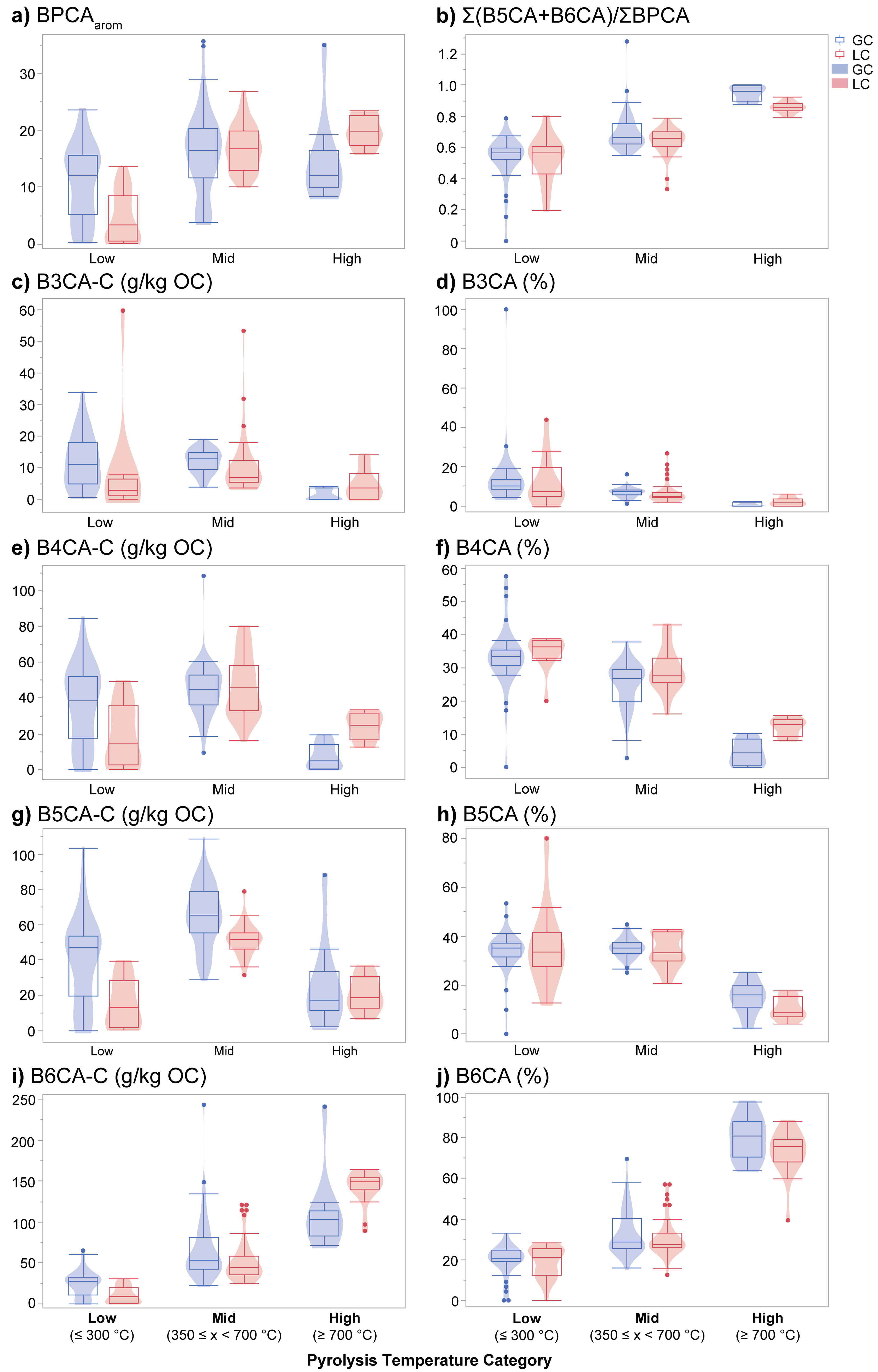

Supplement: S3 Fig — Distribution of GC-obtained (blue) and LC-obtained (red) BPCA results for: a) BPCAarom; b) Σ(B5CA+B6CA)/ΣBPCA; c) B3CA-C (g/kg OC); d) B3CA (%); e) B4CA-C (g/kg OC); f) B4CA (%); g) B5CA-C (g/kg OC); h) B5CA (%); i) B6CA-C (g/kg OC); and j) B6CA (%). Results are separated by pyrolysis temperature category for low (≤ 300 °C), mid (350 ≤ x < 700 °C), and high (≥ 700 °C) temperature chars. All conclusions regarding the statistical significance of differences between the two chromatographic separation methods are relayed in S2 Table (Sheet 5). (TIF) [file pone.0321584.s003.tif]
